# First Case of a Dominant De Novo *SEC23A* Mutation with Neurological and Psychiatric Features: New Insights into Cranio-Lenticulo-Sutural Dysplasia with Literature Review

**DOI:** 10.3390/genes15010130

**Published:** 2024-01-20

**Authors:** Elia Marco Paolo Minale, Alessandro De Falco, Emanuele Agolini, Antonio Novelli, Roberta Russo, Immacolata Andolfo, Achille Iolascon, Carmelo Piscopo

**Affiliations:** 1 U.O.C. Genetica Medica, A.O.U. Federico II, 80131 Naples, Italy; eliaminale@gmail.com (E.M.P.M.); ale.deltafi@gmail.com (A.D.F.); achille.iolascon@unina.it (A.I.); 2 Department of Molecular Medicine and Medical Biotechnology, University Federico II, 80131 Naples, Italy; roberta.russo@unina.it (R.R.); immacolata.andolfo@unina.it (I.A.); 3 Laboratory of Medical Genetics, Ospedale Pediatrico Bambino Gesù, IRCCS, 00165 Rome, Italy; emanuele.agolini@opbg.net (E.A.); antonio.novelli@opbg.net (A.N.); 4 CEINGE Biotecnologie Avanzate, 80145 Naples, Italy; 5 Medical and Laboratory Genetic Unit, Antonio Cardarelli Hospital, 80131 Naples, Italy

**Keywords:** Cranio-lenticulo-sutural dysplasia, *SEC23A*, genotype–phenotype correlation, literature overview, neurological manifestations

## Abstract

Cranio-lenticulo-sutural dysplasia (CLSD, OMIM #607812) is a rare genetic condition characterized by late-closing fontanels, skeletal defects, dysmorphisms, and congenital cataracts that are caused by bi-allelic or monoallelic variants in the *SEC23A* gene. Autosomal recessive inheritance (AR-CLSD) has been extensively documented in several cases with homozygous or compound heterozygous variants in *SEC23A*, whereas autosomal dominant inheritance (AD-CLSD) involving heterozygous inherited variants has been reported just in three patients. The *SEC23A* gene encodes for one of the main components of a protein coat complex known as coat-protein-complex II (COPII), responsible for the generation of the envelope of the vesicles exported from the endoplasmic reticulum (ER) toward the Golgi complex (GC). AR-CLSD and AD-CLSD exhibit common features, although each form also presents distinctive and peculiar characteristics. Herein, we describe a rare case of a 10-year-old boy with a history of an anterior fontanel that closed only at the age of 9. The patient presents with short proportionate stature, low weight, and neurological impairment, including intellectual disability, global developmental delay, abnormal coordination, dystonia, and motor tics, along with dysmorphisms such as a wide anterior fontanel, hypertelorism, frontal bossing, broad nose, high-arched palate, and micrognathia. Trio clinical exome was performed, and a *de novo* heterozygous missense variant in *SEC23A* (p.Arg716Cys) was identified. This is the first reported case of CLSD caused by a *de novo* heterozygous missense variant in *SEC23A* presenting specific neurological manifestations never described before. For the first time, we have conducted a comprehensive phenotype–genotype correlation using data from our patient and the eight most well-documented cases in the literature. Our work has allowed us to identify the main specific and characteristic signs of both forms of CLSD (AR-CLSD, AD CLSD), offering valuable insights that can guide physicians in the diagnostic process. Notably, detailed descriptions of neurological features such as intellectual disability, global developmental delay, and motor impairment have not been documented before. Furthermore, our literature overview is crucial in the current landscape of CLSD due to the absence of guidelines for the clinical diagnosis and proper follow-up of these patients, especially during childhood.

## 1. Introduction

Cranio-lenticulo-sutural dysplasia (CLSD, OMIM #607812) is a rare genetic condition characterized by large and late-closing fontanels, skeletal defects, dysmorphisms, and congenital cataracts that can be inherited in a recessive or dominant pattern [1]. The first clinical description of this condition was made in 2003 in a Saudi family. The patients presented craniofacial features, including wide-open calvarial sutures with large and late-closing anterior fontanel, frontal bossing, hyperpigmentation with capillary hemangioma of the forehead, hypertelorism, and Y-shaped sutural cataracts diagnosed by 1 year of age [1]. Using a positional cloning approach, the homozygous c.1144T>C, p.Phe382Leu variant in *SEC23A* was identified. It was discovered that this mutation segregates with the CLSD phenotype, configuring an autosomal recessive manner of inheritance (AR-CLSD) [2]. *SEC23A* encodes for one of the main components of a protein coat complex known as coat-protein-complex II (COPII). Most of the sequential assembly of the COPII machinery has been well-defined in yeast, where five proteins (Sar1, Sec23, Sec24, Sec13, Sec31) have been identified as the main components. Mammalian orthologues have been identified for each of the five core COPII components, and multiple isoforms of these proteins exist, each encoded by a different gene [3]. The complex is responsible for the formation of vesicle envelopes from the endoplasmic reticulum (ER) to the Golgi complex (GC) [4,5]. Pathogenic variants in *SEC23A* directly impair vesicle formation, leading to the accumulation of secretory material (procollagen1, precursor of COL1A1, the major extra-cellular component of bones), that distend the ER of patient fibroblasts, and this results in an altered network with the GC. In addition to the secretory defect, this accumulation of proteins inside the ER triggers the unfolded protein response and subsequent ER stress, a mechanism underlying various pathologies, including CLSD [6].

Over the years, additional patients with variants in *SEC23A* have been reported. In 2011, a patient with some typical clinical features of CLSD was described. This patient carried only the heterozygous c.2104A>G, p.Met702Val variant in *SEC23A*, which was inherited from the unaffected father [7]. This molecular result has led to three different hypotheses: (1) the possibility that the proband could harbor another variant in a portion of *SEC23A* gene that is undetectable due to low coverage detection rate (e.g., an intronic region); (2) the suspicion of digenic inheritance since the patient had a missense variant in *SEC23A* and potentially a second mutation in another COPII subunit or cargo molecule; (3) the suggestion that CLSD might also have an autosomal dominant inheritance with incomplete penetrance beyond the known AR-CLSD.

In 2022, a 2-month-old baby with large fontanels, wide cranial sutures, large forehead, hypertelorism, thin nose, high-arched palate, and micrognathia was described. A trio whole-genome sequencing (WGS) analysis revealed a heterozygous c.1795G>A, p.Glu599Lys variant in *SEC23A* inherited from the affected father who had dysmorphisms and a history of large and late-closed fontanels [4]. This case further supported the previously proposed autosomal dominant inheritance hypothesis, thereby defining the autosomal dominant form of CLSD (AD-CLSD). Among the AD-CLSD, even though there are few cases, there is high variable expressivity [4] and reduced penetrance [7] in parental inherited cases.

Herein, we report a 10-year-old boy who has been followed by our genetic unit since he was 5 months old. He exhibits the clinical and phenotypic manifestations of CLSD, involving also atypical neurological and neuropsychiatric manifestations. Trio whole-exome sequencing (WES) analysis revealed a *de novo* heterozygous variant in *SEC23A*, previously undescribed, indicating that our patient was affected by AD-CLSD. Furthermore, we have collected the well-described cases of AR-CLSD and AD-CLSD from the literature and made genotype–phenotype correlations for these two forms to better understand their similarities and differences. Through this, we propose a baseline monitoring protocol to guide physicians in the diagnostic process and to establish a starting point for the scientific community to develop specific guidelines for the follow-up of patients affected by CLSD.

## 2. Materials and Methods

Clinical investigations and genetic analyses were conducted in accordance with the principles of the Declaration of Helsinki. Ethical review and approval were waived for this study, dealing with a case report conducted according to clinical practice guidelines. Written informed consent has been approved from the patient and his parents to publish this paper.

After obtaining informed consent from the parents of the patient for the genetic testing, peripheral blood samples were collected from the affected subject and the unaffected parents, and genomic DNA was extracted from circulating leukocytes. Enrichment and parallel sequencing were then performed utilizing a custom clinical exome panel, containing more than 8500 genes. Library preparation was carried out by using the Twist enrichment kit, according to the manufacturer’s protocol (Twist Bioscience, South San Francisco, CA, USA), and sequenced on a NovaSeq 6000 (Illumina, Inc., San Diego, CA, USA) platform. The BaseSpace pipeline (Illumina, https://basespace.illumina.com/, accessed on 21 December 2023) and the Geneyx Analysis (AI-driven NGS Analysis platform) formerly known as TGx Gene Cards were used for the variant calling and annotating variants, respectively. Sequencing data were aligned to the hg19 human reference genome. The variants were analyzed in silico by using Combined Annotation Dependent Depletion (CADD) V.1.3 [8], Sorting Intolerant from Tolerant (SIFT) [9], Polymorphism Phenotyping v2 (PolyPhen-2) [10], and Mutation Taster [11] for the prediction of deleterious non-synonymous SNVs for human diseases. In summary, the pathogenicity of each variant was evaluated by gathering evidence from the above sources, including population data, computational and predictive data, and segregation data, in accordance with the guidelines of the American College of Medical Genetics and Genomics (ACMG) [12]. Variants were examined for coverage and Qscore (minimum threshold of 30) and visualized by the Integrative Genome Viewer (IGV). The prioritized variant was confirmed by Sanger sequencing and by analysis of inheritance patterns.

## 3. Results

### 3.1. Proband Phenotype

**Figure 1:**
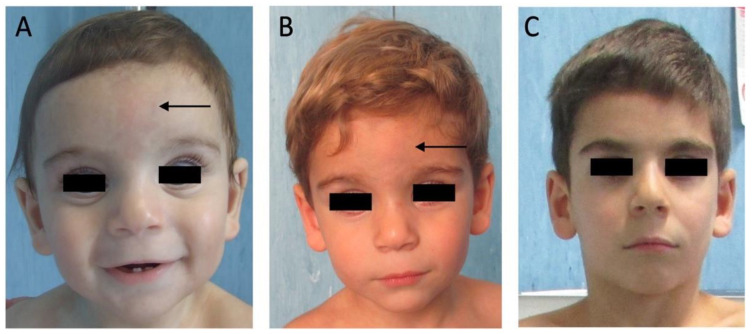
Photo of our proband, taken at different ages: 1 year and 11 months old (**A**), 3 years and 4 months old (**B**), and 7 years and 5 months old (**C**). It is possible to appreciate distinctive phenotypic features: large forehead, frontal bossing, hypertelorism, a broad nose, a high-arched palate, thin upper lip, micrognathia, and a long, smooth philtrum. Moreover, it is possible to see skin hyperpigmentation with capillary hemangioma of the forehead along the metopic suture, as indicated by the black arrow in (**A**,**B**).

Our patient is the second child of healthy, non-related parents. The first child is in good health and shows no signs of dysmorphic features, neurodevelopmental delay, intellectual disability, short stature, or other health problems. The proband was born at 38 weeks of gestation. At birth, weight was 2450 g (−2.51 SD), length 48 cm (8° pc; −1.38 SD), occipital-frontal circumference (OFC) 34 cm (22° pc; −0.78 SD) and he had an Apgar Score of 8 at the first minute and 9 after five minutes. Low-set ears and hypertelorism were observed during the initial clinical examination at birth. Ten days after birth, he exhibited motor hyperexcitability with tremors, leading to hospitalization for further investigation. The diagnostic workup included a comprehensive ophthalmological examination, including fundus oculi (which showed low-grade hyperopia and myopia), echocardiography (revealing interatrial and subaortic ventricular defects), brain MRI, sleep EEG, ABR, Visual Evoked Potential (VEP) tests, thyroid profiling, and aminoacidic screening, all of which yielded normal results. At 5 months of age, he was referred to our genetic unit for a complete physical examination. His weight was 4.9 kg (<1° percentile; −3 SD), length 59 cm (<1° percentile; −2.6 SD) and OFC 40.5 cm (4° percentile). He exhibited an open bregmatic fontanel, skin hyperpigmentation with capillary hemangioma of the forehead and dysmorphisms like large forehead, frontal bossing, hypertelorism, broad nose, high-arched palate, micrognathia, and long smooth philtrum (Figure 1A–C).

**Figure 2:**
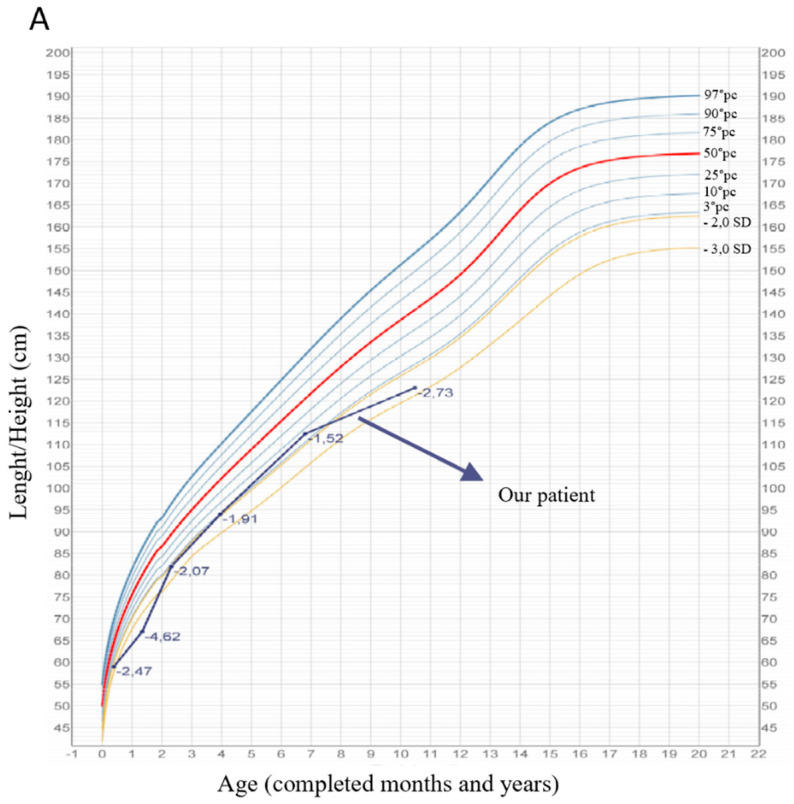

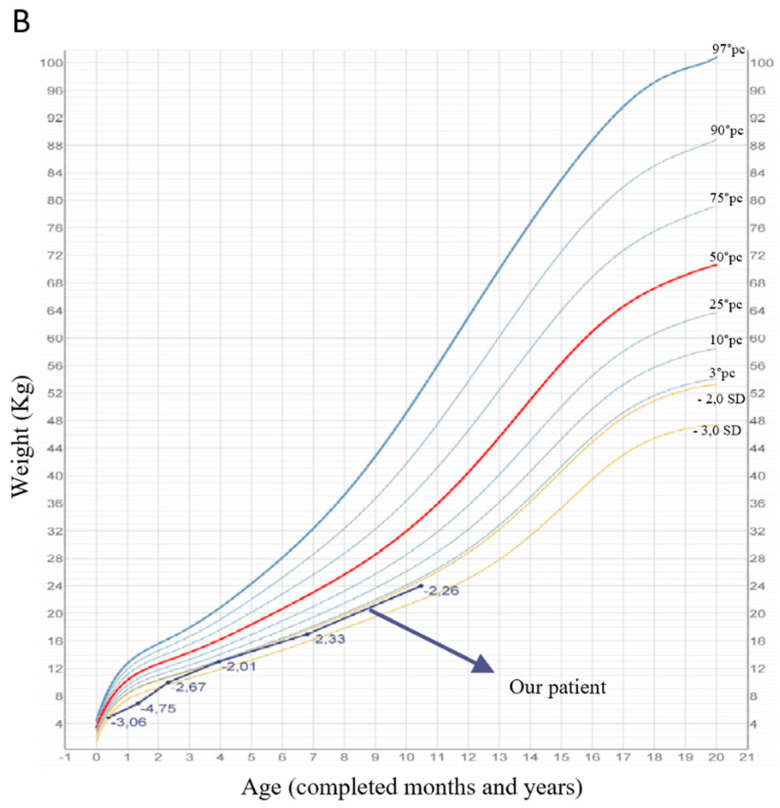
Growth charts for the length/height (**A**) and weight (**B**) of our patient referring to CDC, 2002 [13]. From the charts over time, it is possible to observe both stature and weight below the 3rd percentile [made with Growth Calculator 3.0 Software—Società Italiana di Endocrinologia e Diabetologia Pediatrica].

At the age of 10 months, the patient underwent surgical correction for cryptorchidism and unilateral inguinal hernia. We have conducted periodic patient assessments up to the age of 10 years, in many of which we consistently observed poor growth in height and weight (both below the third percentile) (Figure 2), while the OFC remained within normal limits. The height of his father is 178 cm while the height of his mother is 168 cm so the genetic target for the height of our patient is between 168.5 and 185.5 (10°–90° percentiles): our patient has short stature which is outside the parental genetic target.

The patient underwent an endocrinological assessment with GH and IGF1 evaluation, which yielded normal results. During these periodic assessments, we have noted the closure of the bregmatic fontanel at the age of 9 years, along with motor impairment and neurodevelopmental delays which started to be observed from the age of 2 years. Indeed, when he was 31 months old, global developmental delay was diagnosed by applying the Bayley III scale, yielding an average score of approximately the 1.43° percentile. More specifically, the cognitive and language scales both scored at the 2° percentile, while the motor scale scored at the 0.3° percentile. At 10 years old, a neuropsychiatric evaluation was conducted using the WISC-IV rating scale, resulting in a global IQ score of 40, a General Ability Index (GAI) of 46, and a Cognitive Proficiency Index (CPI) of 42, leading to the diagnosis of intellectual disability. The last neurological examination at 10 years of age revealed motor clumsiness with frequent dystonic movements and facial motor tics. In the suspicion of a rare genetic disorder, singleton array-CGH was performed, but it yielded normal results. Thus, a trio clinical exome was carried out.

### 3.2. Exome Sequencing and Variant Annotation

Trio clinical exome analysis revealed the *de novo* heterozygous missense variant c.2146C>T, p.Arg716Cys in the SEC23A gene (NM_006364.4), which is associated with CLSD. We also searched for the same variant in the older brother, which was not found. The variant p.Arg716Cys, confirmed by Sanger sequencing analysis, was not previously described in the scientific literature, nor was it annotated in gnomAD [11] or 1000G [14] (Table 1) but was predicted to be highly pathogenic by CADD-based prediction [8]. It has been reported in ClinVar (RCV001589365) [15] but there is no well-described information, and the variant was not segregated in the proband’s family. Computational prediction tools, such as MetaRN, support its deleterious effect on the gene (MetaRN score: 0.922) [16]. The *de novo* nature of the variant and its damaging effect on the gene, in a patient with a consistent phenotype and no family history, led to its interpretation as a likely pathogenic variant, according to the ACMG criteria (PM2, PP3, PP4, PS2 criteria) [12].

Additionally, the trio clinical exome revealed the presence of the heterozygous variant c.3034G>A, p.Ala1012Thr in the TNIK gene in the proband inherited from the unaffected mother. According to ACMG criteria, this variant has been classified as a variant of uncertain significance (PM2, PP2, BP4 criteria). Pathogenic variants in this gene are associated with “intellectual developmental disorder, autosomal recessive 54” (OMIM #617028), primarily discovered in consanguineous families [17]. Since this condition has only autosomal recessive inheritance, and our patient has only one variant of uncertain significance, we have considered this variant not to be responsible for the features presented by our patient.

### 3.3. Genotype–Phenotype Correlation

In order to provide a comprehensive and appropriate comparison between the clinical characteristics of AD-CLSD and AR-CLSD, we have reviewed the literature, selecting articles where the CLSD patients were completely described both molecularly and clinically: in this way, we have collected the clinical and molecular data of five patients affected by AR-CLSD and four patients affected by AD-CLSD (including our proband) for a total of nine patients (Table 2).

Among the eight cases already reported in the literature with mutations in the *SEC23A* gene, four patients were described with the homozygous missense variant p.Phe382Leu [1], one patient harbored the heterozygous missense variant p.Met702Val [7], two patients had the heterozygous missense variant p.Glu599Lys [4], and one patient harbored the two missense variants p.Asp237Ala and p.Leu649Pro in compound heterozygosity [18]. We have grouped the main features of these nine patients (the eight patients present in the literature and our patient) into five groups according to phenotypic and neuro-psychiatric criteria using the standardized terms of Human Phenotype (HP) Ontology [19]: head abnormalities (HP:0000234), eye abnormalities (HP:0000478), facial abnormalities (HP:0000271), growth delay (HP:0001510), and abnormalities in nervous system function (HP:0012638).

Regarding craniofacial abnormalities, all patients (9/9, 100%) exhibit a wide anterior fontanel (HP:0000260) with delayed closure (HP:0001476), which is a hallmark feature of the syndrome. All patients with AR-CLSD (5/5, 100%) exhibit skin hyperpigmentation with capillary hemangioma on the forehead, whereas in the case of AD-CLSD, this sign is exclusively present in our patient. We do not know if this sign has been assessed in the other reported cases of AD-CLSD. However, based on our periodic evaluations in our patient, we can assert that this characteristic feature tends to regress between the age of 3 and 7 until it disappears, as can be observed in Figure 1C.

About the eye abnormalities, hypertelorism is consistently observed in our entire cohort (9/9, 100%), bilateral cataracts (HP:0000518) is present in all the patients affected by AR-CLSD but it is not present in AD-CLSD patients: this is a peculiar difference between the two forms of inheritance, identifying a distinctive and typical finding of AR-CLSD and allowing an important clue to make differential diagnosis between the two different inherited CLSD.

Dysmorphisms are also a prominent aspect of this syndrome, with frontal bossing (HP:0002007) being prevalent in 100% of our cohort (9/9). Additionally, abnormal nasal dorsum morphology (HP:0011119), in particular broad nasal bridge and anteverted nares, is present in 88% of our cohort (5/5, 100% of AR-CLSD; 3/4, 75% in AD-CLSD), abnormal upper lip morphology (HP:0000177), particularly thin upper lip, is observed in 77% of our cohort (5/5, 100% of AR-CLSD; 2/4, 50% of AD-CLSD), while arched palate (HP:0000218) and midface retrusion (HP:0011800) are both found in 66% of the cohort but while the former is present in 3/5 (60%) of AR-CLSD and 3/4 (75%) of AD-CLSD, the latter is present in 4/5 (80%) of AR-CLSD and in 2/4 (50%) of AD-CLSD. Downslanted palpebral fissures are absent from the AR-CLSD form, while they are present in 2/4 (50%) of AD-CLSD.

Concerning growth delay, proportionate short stature (HP:0003508) is observed in all the patients except for patient 5 because the authors focused on the ophthalmologic aspects and not on weight–stature balance [18]. The presence of growth delay needs to be meticulously evaluated and monitored by clinicians who approach children affected by CLSD. Regarding the abnormalities in nervous system function, delayed speech, and language development (HP:0000750), motor delay (HP:0001270) and global developmental delay (HP:0001263) have been previously observed in the literature [1,7]. Global development delay is present in 2/5 (40%) patients with AR-CLSD and 2/4 (50%) of AD-CLSD for a total of 4/9 (44%) in CLSD.

Differently, our patient exhibits distinctive neuropsychiatric and neurological features that have not been previously described and considered as associated with this condition: intellectual disability (HP:0001249), coordination abnormalities (HP:0011443), dystonia (HP:0001332), and motor tics of the face (HP:0100034).

## 4. Discussion

Herein, we report clinical and genetic findings of a male patient with AD-CLSD. The molecular and clinical features of our patient confirm the main characteristics of AD-CLSD, including a wide anterior fontanel with delayed closure, skeletal defects (such as short stature), and dysmorphism (hypertelorism, thin upper lip, frontal bossing, long smooth philtrum, broad nasal bridge, arched palate). Additionally, our patient presents neuropsychiatric and neurological features, including global developmental delay, intellectual disability, motor coordination disorder with frequent dystonic movements, and motor tics of the face.

We have hypothesized that the peculiar and distinctive features of the case herein described may be linked to the tissue-specific effects of the newly identified missense variant p.Arg716Cys in the *SEC23A* gene. Indeed, the analysis of the clinical features presented in our table reveals that certain tissues are more affected than others in both inherited forms. The key aspect of tissue-specific manifestations can be attributed to reduced *SEC23A* activity in CLSD-affected patients, which can be sufficient for some cells but inadequate for others, particularly those with a high rate of secretory exocytic pathway activity.

SEC23A is fundamental in cytoskeletal signaling [20]. During fetal cranial development, this signaling is connected to SHH pathway: deregulation of this signaling with hyperregulation of GLI transcription factors could lead to face and eye abnormalities of the *SEC23A* phenotypes [21].

Furthermore, in vitro studies have indicated that an elevated level of SAR1B (SAR1 paralog) relative to SAR1A (SAR1 paralog) might exacerbate the defect in specific cell types: there is a suspicion that SEC23B protein, a mammalian paralog of SEC23A, may play a compensatory role to supplement SEC23A function [22]. Notably, the functional overlap between the two SEC23 paralogs has been already demonstrated in different human compartments, such as erythroid cells and hepatic cells [23,24]. Therefore, we could suppose that the tissues most affected in CLSD are susceptible to variations in SEC23A levels or may have an insufficient expression of SEC23B or elevated SAR1B expression. Further RNA sequencing (RNAseq) or whole transcriptome analysis (WTA) studies in different tissues of CLSD patients could support this hypothesis: WES combined with RNAseq/WTA could help us to better understand the underlined physiopathology [25].

It is also interesting to note that all the *SEC23A* variants described so far in the literature, including the variant described in our patient, are missense variants. This suggests that missense variants may play a pivotal role in the pathogenesis of CLSD, influencing the structure and, subsequently, the function of SEC23A, impacting its interaction with other proteins within the COPII complex. Moreover, in AR-CLSD forms, the variants in *SEC23A* affect the early exons of the gene (except for patient 5 who is a compound heterozygous [18]) while, in AD-CLSD forms, they are present in the last exons of the gene. This clustering of variants, which occurs differently according to the inheritance of CLSD, suggests that the *SEC23A* gene could account for two different forms of syndrome with common features but different clinical outcomes. It should be interesting to focus on these clustering variants and to study the episignature of this gene: we expect two different episignatures for *SEC23A*, according to the pattern of inheritance [26].

To support the hypothesis that neurological symptoms may be linked to the pathophysiology of CLSD and considering that the symptoms described in our patient have not been reported before in patients with CLSD, we have searched for syndromes that share defective ER–Golgi network/ER-stress, a similar pathophysiological mechanism with CLSD. Examples include Halperin–Birk syndrome (OMIM #618651) and Wolfram syndrome type 1 (OMIM #222300), both of which also exhibit neurological and psychiatric manifestations. Halperin–Birk syndrome is caused by biallelic mutations in the *SEC31A* gene, another main component of COPII. These gene alterations cause a molecular pathogenesis comparable to CLSD, with COPII dysfunction, defects in procollagen secretion, subsequent enlargement of ER (ER-stress), and aberrant protein secretion (defective ER–Golgi network). Notably, this syndrome shares some clinical features with CLSD, including skull deformities, micrognathia, high-arched palate, and bilateral congenital cataracts. It is important to note that the main features of Halperin–Birk syndrome include intellectual disability and developmental delay, which are also present in our patient [27]. However, this syndrome is more severe than CLSD, because it presents spastic quadriplegia with multiple contractures, seizures, and pseudobulbar palsy. Generally, other conditions, which have defective ER–Golgi network as their physiopathological basis, like Wolfram syndrome, also exhibit neurological and psychiatric alterations [28]. From these observations, it can be deducible that genetic defects leading to ER stress can generally cause neurological and psychiatric abnormalities, as reported in the literature [6]. Specifically, COPII complex abnormalities are associated with intellectual disability, developmental delay, and other neurological issues, as seen in our patient. It would be challenging to collect more clinical data from the already described patients in the literature, to observe their evolution over the time, and to focus also on the neurological manifestations.

## 5. Conclusions

We present the case of a 10-year-old boy with wide anterior fontanel with delayed closure, dysmorphisms, and short proportionate stature. Genetic analysis revealed the *de novo* heterozygous missense variant p.Arg716Cys in the *SEC23A* gene and so this is the first reported case of AD-CLSD presenting specific neurological manifestations never described before.

To better understand the similarities and differences between AR-CLSD and AD-CLSD manifestations, we have performed a genotype–phenotype correlation of nine patients (eight from the literature and our patient). This analysis reveals that all nine share common features, independent of the inheritance pattern, such as a wide anterior fontanel with delayed closure, hypertelorism, frontal bossing, and proportionate short stature. Notably, bilateral cataracts, which emerges early in life, may serve as a specific indicator of AR-CLSD.

Another significant finding is the presence of distinct neuropsychiatric and neurological features (intellectual disability, coordination abnormalities with dystonia, and facial motor tics) which are observed in our patient, but they have been previously unreported in CLSD. The underlying causes of the emergence of neurological and neuropsychiatric symptoms in CLSD represent an area that warrants further investigation within the scientific community.

Finally, the information produced by our genotype–phenotype correlation could offer valuable insights in the diagnostic process. This is useful in the current landscape of CLSD due to the lack of guidelines for the proper diagnosis and follow-up of these patients, especially during childhood. In fact, we propose early ophthalmological evaluations for AR-CLSD patients, periodic monitoring of growth patterns, and screening for neuropsychiatric and neurological features through specific diagnostic tools/exams in both inherited forms of CLSD. We believe that our proposed screening methods will not only help in identifying but also in better characterizing both form of CLSD, thus advancing our understanding of the symptoms of it and improving the care of the patients.

## Figures and Tables

**Table 1:** Annotation features of NM_006364.4: c.2146C>T variant.

**Gene Symbol**	*SEC23A*
**Genomic location (hg19)**	chr14-39508297 G>A
**HGVS nomenclature cDNA-level**	NM_006364.4: c.2146C>T
**HGVS nomenclature Protein-level**	NP_006355.2: p.Arg716Cys
**Exon**	19/20
**MetaRN**	0.922
**CADD Score**	28.1
**Allele Frequency (gnomAD)**	N/A
**Allele Frequency (1000G)**	N/A

**Table 2:** Clinical characteristics of affected individuals with *SEC23A* mutations present in the literature and divided into AR-CLSD and AD-CLSD. Calculation of the percentage of the five patients affected by AR-CLSD and of the four patients affected by AD-CLSD and recalculation of the new percentages of the features of CLSD in general. The grey column stands for our index patient. “+” stands for clinical features of CLSD presented in the case reported, while “–” stands for clinical features of CLSD not presented. NA: not available; TOT.: total.

Inheritance Pattern		AR-CLSD						AD-CLSD					CLSD
Patients		1	2	3	4	5	TOT	6	7	8	9	TOT: 4 (%)	TOT: 9 (%)
Literature		Boyadjiev, 2003 [1]				Wang, 2023 [18]		Boyadjiev, 2011 [7]	Cisarova, 2022 [4]		Our Patient		
**Protein change(s) in the *SEC23A* gene**		p.Phe382Leu; p.Phe382Leu	p.Phe382Leu; p.Phe382Leu	p.Phe382Leu; p.Phe382Leu	p.Phe382Leu; p.Phe382Leu	p.Asp237Ala; p.Leu649Pro		p.Met702Val	p.Glu599Lys	p.Glu599Lys	p.Arg716Cys		
**Exon**		10/20	10/20	10/20	10/20	7/20–17/20		18/20	16/20	16/20	19/20		
**Head abnormalities**	Wide anterior fontanel with delayed closure	+	+	+	+	+	5/5 (100%)	+	+	+	+	4/4 (100%)	**9/9 (100%)**
**Eye abnormalities**	Bilateral cataracts	+	+	+	+	+	5/5 (100%)	−	−	−	−	0/4 (0%)	**5/9 (55%)**
	Hypertelorism	+	+	+	+	+	5/5 (100%)	+	+	+	+	4/4 (100%)	**9/9 (100%)**
**Facial abnormalities**	Frontal bossing	+	+	+	+	+	5/5 (100%)	+	+	+	+	4/4 (100%)	**9/9 (100%)**
	Skin hyperpigmentation with capillary hemangioma of the forehead	+	+	+	+	+	5/5 (100%)	NA	NA	NA	+	1/1 (100%)	**6/6 (100%)**
	Downslanted palpebral fissures	−	−	−	−	−	0/5 (0%)	+	+	−	−	2/4 (50%)	**2/9 (33%)**
	Abnormal nasal dorsum morphology	+	+	+	+	+	5/5 (100%)	+	+	−	+	3/4 (75%)	**8/9 (88%)**
	Abnormal upper lip morphology	+	+	+	+	+	5/5 (100%)	−	−	+	+	2/4 (50%)	**7/9 (77%)**
	Arched palate	+	+	−	−	+	3/5 (60%)	+	+	−	+	3/4 (75%)	**6/9 (66%)**
	Midface retrusuion	+	+	+	+	−	4/5 (80%)	−	+	−	+	2/4 (50%)	**6/9 (66%)**
**Growth delay**	Proportionate short stature	+	+	+	+	NA	4/4 (100%)	+	+	+	+	4/4 (100%)	**8/8 (100%)**
**Abnormalities in nervous system function**	Global developmental delay	−	−	−	+	+	2/5 (40%)	+	−	−	+	2/4 (50%)	**4/9 (44%)**
	Intellectual disability	−	−	−	−	−	0/5 (0%)	−	−	−	+	1/4 (25%)	**1/9 (11%)**
	Abnormality of coordination	−	−	−	−	−	0/5 (0%)	−	−	−	+	1/4 (25%)	**1/9 (11%)**
	Dystonia	−	−	−	−	−	0/5 (0%)	−	−	−	+	1/4 (25%)	**1/9 (11%)**
	Motor tics	−	−	−	−	−	0/5 (0%)	−	−	−	+	1/4 (25%)	**1/9 (11%)**

## Data Availability

Data are contained within the article.
